# Breastfeeding and risk of childhood CNS tumours

**DOI:** 10.1038/sj.bjc.6603638

**Published:** 2007-03-06

**Authors:** N J Harding, J M Birch, S J Hepworth, P A McKinney

**Affiliations:** 1 Paediatric Epidemiology Group, 30-32 Hyde Terrace, University of Leeds, Leeds LS2 9LN, UK; 2 Cancer Research UK Paediatric and Familial Cancer Research Group, Central Manchester and Manchester Children's University Hospitals NHS Trust, Manchester, M274HA, UK

**Keywords:** breast feeding, childhood cancer, CNS tumours, case control study

## Abstract

We investigated infant feeding habits in relation to risk of childhood central nervous system tumours among 633 cases in the UK Childhood Cancer Study (UKCCS). No significant effect of breastfeeding was detected overall (odds ratio 1.01, confidence interval: 0.85–1.21) nor in any morphological subgroup. Similarly, no effect for the duration of breastfeeding or any other feeding practices was observed.

Central nervous system (CNS) tumours are the second most common group of childhood cancers after leukaemia. Rare genetic disorders can predispose children to a small proportion of CNS tumours, (Bondy, 1991; Little, 1999) but attempts to identify underlying environmental risk factors have largely been unsuccessful, with only ionising radiation known to confer an increased risk (Linet *et al*, 2003).

Childhood leukaemia may have an infectious aetiology (McNally and Eden, 2004) but reported links between childhood leukaemia and breastfeeding (reviewed by Guise *et al*, 2005) are inconsistent (Dockerty *et al*, 1999; Shu *et al*, 1999; Rosenbaum *et al*, 2000; Beral *et al*, 2001).

Interest in a link between childhood CNS tumours and infections has arisen from epidemiological analyses (Nyari *et al*, 2003; Altieri *et al*, 2006; Shaw *et al*, 2006) and excess space–time clustering and seasonality of cases (McNally *et al*, 2002), though not all these findings have been replicated (McNally *et al*, 2004). There is an apparent dearth of studies investigating the effect of breastfeeding on childhood CNS tumours. Schuz *et al* (2001) have reported no effect of breastfeeding on the risk of childhood CNS tumours whereas other studies have similar results for a broad group of ‘other cancers’ containing CNS tumours (Mathur *et al*, 1993; Beral *et al*, 2001; Lancashire and Sorahan, 2003). The likely differences in aetiology between CNS cancer types mean any subgroup-specific effect of feeding habits may have been masked. This study examines the effect of feeding habits for all CNS tumours, but also by diagnostic subgroup to address possible differences in aetiology.

## PATIENTS AND METHODS

The UKCCS is a nationwide population-based case–control study of childhood malignancies established with the aim of identifying risk factors for childhood cancer. Details of the study are published elsewhere (UKCCS Investigators, 2000). Briefly, children diagnosed with cancer before 15 years of age were eligible for inclusion between 1991–1994 for all diagnoses in Scotland and 1992–1996 in England and Wales. Cases of solid tumours, including CNS tumours were recruited to 1994. A pathological review provided detailed classification of tumours. Two controls per case were selected at random from health authorities/health boards and matched by birth month/year and study region, non-participating controls were replaced.

Mothers of case and control subjects were interviewed using a questionnaire detailing whether they had ever breastfed, including dates and durations, whether they had ever used formula milk, whether they sterilised bottles and feeding utensils, and the age at which solid food was introduced.

A total of 7621 controls and 686 cases were available for the study. Children under 12 months of age (51 CNS cases, 631 controls) at diagnosis/pseudodiagnosis were excluded to prevent bias caused by premature cessation of breastfeeding owing to cancer. Children were also excluded where the questionnaire was not completed by the biological mother (two cases, 35 controls).

Analyses were carried out for all CNS tumours (*n*=633), and diagnostic subgroups; all gliomas (*n*=347) (including pilocytic astrocytoma (*n*=160)), ependyoma (*n*=65), medulloblastoma/PNET (*n*=149), and other CNS tumours (*n*=72). The comparison control group included all controls from the entire study, a procedure common to all UKCCS studies (Beral *et al*, 2001).

Odds ratios (OR) were estimated using unconditional logistic regression and were adjusted for age (in one year intervals), sex, study region, and Townsend deprivation index (Townsend *et al*, 1988) derived from the residential address at diagnosis. Analyses were undertaken investigating the effect of breastfeeding, sterilising feeding utensils, and the age at which the child was first introduced to solid food.

## RESULTS

Overall, the proportion who had ever been breastfed was very similar between cases and controls (64.1% cases *vs* 63.5% controls), as was the proportion breastfed for over 6 months (26.4 *vs* 26.5%).

ORs and 95% confidence intervals (CIs) for the effect of breastfeeding on CNS tumour risk can be seen in Table 1. No significant associations were observed between ever having breastfed and all CNS tumours or any diagnostic subgroup, nor was there any statistically significant effect of duration of breastfeeding. The OR of developing any CNS tumour is 1.01 (CI: 0.85–1.21) of ever having been breastfed to never having been breastfed, and for over 6 months compared to never is 1.02 (CI: 0.82–1.27).

Mothers of controls who breastfed only did so on average for 11.6 months compared to 3.6 months in mothers who also used formula feed (*t*-test; *P*<0.001). Women who formula fed also introduced their children to solid food earlier at 3.9 months compared to 4.3 months in women who only breastfed (among controls) (*t*-test; *P*<0.01). Breastfeeding habits differed greatly according to Townsend deprivation category; areas exhibiting the highest levels of deprivation showed the lowest level of breastfeeding (*χ*^2^ trend test *P*<0.001; Altman, 1991). Birth order also influenced breastfeeding habits, with later children being less likely to have been breastfed (*χ*^2^ trend test *P*<0.001). Older children were less likely to have been given formula feed (*χ*^2^ trend test, 1 year age intervals, *P*=0.001) but no more likely to have been breastfed (*P*=0.181); it is unclear whether this constitutes bias or a shift in feeding habits.

None of the further analyses of sterilisation or age at introduction of solid food showed a significant effect for all CNS tumours or any diagnostic subgroup (results not shown), although an increased risk associated with sterilising feeding utensils did approach significance (OR 1.54, *P*=0.067, CI: 0.97–2.45). Analyses repeated using a matched design (1251 controls) obtained similar results (results not shown).

## DISCUSSION

Our results provide no evidence to suggest breastfeeding either positively or negatively influences the risk of childhood CNS cancers. No effects of ever breastfeeding or of the duration of breastfeeding were observed. Our findings are consistent with Schuz *et al* (2001) although based on larger numbers and analysed by diagnostic subgroups. However, if CNS tumours do have an infectious aetiology, these results may not translate to developing countries, where the protective effect of breastfeeding against infection is likely to be more significant (WHO Collaborative Study Team, 2000).

Case–control studies are vulnerable to bias (Rothman, 1998) and the UKCCS is subject to participation bias; responding controls are generally from less deprived areas and therefore are not completely representative of the underlying population (Law *et al*, 2002). Areas of higher deprivation display a lower level of breastfeeding (Wright *et al*, 2005); this is also shown in our results. Despite attempts to adjust for deprivation it is possible that a confounding effect may remain. Recall bias is also a potential problem, with the possibility of differential reporting between cases and controls. Self-reporting of breastfeeding habits are known to lack accuracy (DHS Comparative Studies, 1999), though it is unclear whether this differs between cases and controls.

Whether or not the mother had ever sterilised feeding utensils approached significance. This result is driven by differences between case and control mothers who have solely breastfed; owing to multiple comparisons it is possible this has occurred by chance.

In summary, this study found no evidence that breastfeeding and other infant feeding habits influence the risk of childhood CNS tumours.

## Figures and Tables

**Table 1:** Numbers of subjects (*n*) and ORs for association between breastfeeding and childhood CNS tumours by diagnostic group

				**Ever breastfed (duration)**				
	**Exposure**	**Never breastfed**	**Ever breastfed**	**<1 month**	**1–6 months**	**>6 months**	**Unknown**	***P*-value for trend** **
Controls	*n* (%)	2495 (35.9)	4460 (64.1)	1014 (14.6)	1599 (23.0)	1842 (26.5)	5	
								
All CNS tumours	*n* (%)	231 (36.5)	402 (63.5)	101 (16.0)	134 (21.2)	167 (26.4)	0	0.72
	OR (95% Cl)	1.00	1.01 (0.85–1.21)	1.11 (0.86–1.42)	0.94 (0.75–1.19)	1.03 (0.83–1.28)		
								
Glioma	*n* (%)	122 (35.2)	225 (64.8)	55 (15.9)	70 (20.2)	100 (28.8)	0	0.59
	OR (95% Cl)	1.00	1.08 (0.86–1.38)	1.14 (0.82–1.60)	0.95 (0.70–1.30)	1.19 (0.89–1.58)		
								
Pilocytic astrocytoma^a^	*n* (%)	67 (41.9)	93 (58.1)	27 (16.9)	29 (18.1)	37 (23.1)	0	0.17
	OR (95% Cl)	1.00	0.82 (0.59–1.15)	1.02 (0.64–1.61)	0.71 (0.45–1.13)	0.80 (0.52–1.23)		
								
Ependyoma	*n* (%)	23 (35.4)	42 (64.6)	13 (20.0)	11 (16.9)	18 (27.7)	0	0.77
	OR (95% Cl)	1.00	1.01 (0.59–1.73)	1.41 (0.70–2.82)	0.72 (0.35–1.51)	1.03 (0.54–2.00)		
								
Medulloblastoma/PNET	*n* (%)	53 (35.6)	96 (64.4)	25 (16.8)	36 (24.2)	35 (23.5)	0	0.61
	OR (95% Cl)	1.00	1.01 (0.71–1.45)	1.16 (0.71–1.89)	1.07 (0.69–1.67)	0.88 (0.56–1.37)		
								
Other CNS tumours	*n* (%)	33 (45.8)	39 (54.2)	8 (11.1)	17 (23.6)	14 (19.4)	0	0.22
	OR (95% Cl)	1.00	0.77 (0.47–1.25)	0.65 (0.30–1.42)	0.89 (0.49–1.64)	0.69 (0.36–1.34)		

CNS=central nervous system; OR=odds ratio.

a Subgroup of glioma.

** *P*-value derived from fitting a linear trend across categories in a logistic regression model.

Logistic regression analyses adjusted for age, sex, region, and deprivation index.
